# Analysis of COVID-19 outbreak origin in China in 2019 using differentiation method for unusual epidemiological events

**DOI:** 10.1515/med-2021-0305

**Published:** 2021-06-28

**Authors:** Vladan Radosavljevic

**Affiliations:** Institute of Epidemiology, Military Medical Academy, Crnotravska 17, 11000 Belgrade, Serbia

**Keywords:** Outbreak, pandemic, COVID-19, outbreak origin, outbreak differentiating, SARS-CoV-2

## Abstract

**Objectives:**

Origin of outbreaks could be natural, accidental, deliberate, and caused by a new or re-emerging bioagent. The aim of this study was the retrospective analysis of whether the COVID-19 outbreak was natural, accidental, deliberate one, or caused by a new or re-emerging bioagent.

**Methods:**

Analysis was performed according to the Radosavljevic–Belojevic method for outbreak scoring and differentiation. Data for the application of this method were obtained by literature review in the Medline database for the period from 2000 to 2020.

**Results:**

The analysis of the unusual COVID-19 outbreak shows that the present official assumption of its natural origin is questionable and pointed out to a probability that the pathogen could have also been accidentally introduced in the human population.

**Conclusion:**

There are no conclusive pieces of evidence about the reservoir of the pathogen or the source of infection. These parameters are essential for the final clarification of the outbreak origin. This study suggests that the COVID-19 outbreak is a consequence of an accidental release of a new COVID-19 virus, probably during the technical accident and/or negligent violation of hygienic norms in the laboratory facility. Further epidemiological, microbiological, and forensic analyses are needed to clarify the COVID-19 outbreak.

## 1 Introduction

The origin of the COVID-19 pandemic is of crucial importance because of the following reasons: over three million people have died worldwide, hundreds of millions of people got infected and diseased, psychological consequences on people, and enormous economic losses. The first postulate of the outbreak investigation is to find out the source/reservoir of the outbreak and eliminate it to stop epidemic expansion, and hence the explicit and detailed description of the origin of COVID-19 pandemic is needed. For a year and a half, along with common China and WHO investigation, and hundreds of published scientific articles, we do not yet have any conclusive answer about the origin of the COVID-19 pandemic. So, the reliable and appropriate method for detection of the pandemic origin has to be applied.

The origin of outbreaks could be natural, accidental, deliberate, and caused by a new or re-emerging bioagent [1]. There are very important issues/questions about COVID-19 outbreak origin:

(1) What is the source of the outbreak?
(2) Is it possible to prove an animal(s) to be the source/reservoir of the outbreak?
(3) Is there proof of infection/disease in humans with the COVID-19 virus directly transmitted from animals?
(4) Were there dead animals infected with the COVID-19 virus?
(5) Was there a reverse spread with the COVID-19 virus (from humans to animals)?
(6) Epidemiologists first look for the source of the outbreak to eliminate or mitigate it. In the COVID-19 outbreak, there is no scientific information about such or similar attempts in China (country of origin). Instead, there were stories about N.N. person(s) who visited Huanan Seafood Wholesale Market and made pandemic, and “TV introduction” with the fauna of Southeastern Asia (bats, pangolins, snakes, and turtles). Should we believe it?
(7) Does this outbreak originate as a onetime infection or as a repeated infection?
(8) How did the infectious dose start the outbreak? Was the infectious dose from a couple of heat-treated bats enough?
(9) What happened with the bat hunters and people who prepared and transported the “infected bats” to the market? [2]
(10) It takes weeks, months, and in some cases years for establishing the complete genome. Chinese authorities officially informed WHO on December 31, 2019 about the COVID-19 outbreak and as of January 12, 2020 sent the complete genome sequence worldwide. It looks strange that Chinese scientists detect a complete genome sequence within a couple of weeks without any previous contact with the COVID-19 virus.
(11) Luc Montagnier publicly announced that the genome sequence was artificially changed. There is no serious scientific denial of it.
(12) Chinese official data of less than 5000 deaths seem unbelievable? Such data should be compared [3] with data from other countries.
(13) If Chinese anti-epidemic measures are so effective, why did not the other countries incorporate them?
(14) What experiments did scientists in Wuhan Institute of Virology perform on bats? [4]
(15) Were preventive measures regarding staff behavior and use of equipment in Wuhan Institute of Virology satisfactory?
(16) Although Chinese experts were introduced very well with a set of 19 questions that were prepared for early and distant outbreak differentiation, those questions have not been mentioned until now [5].
(17) Chinese officials took too much time to publicly report the unusual/atypical manifestation (heavily moribund patients with fulminant illness course) of a known disease (pneumonia) [6].
(18) Chinese authorities did not report the existence of several unusual/unexplained syndromes (loss of smell and taste) coexisting in the COVID-19 patients.
(19) Strangely, Chinese public health authorities did not report for 1 month (December 2019) a sudden unexplainable increase in the morbidity and/or mortality rates in human population (higher than expected) for pneumonia [6].
(20) Chinese medical authorities did not report for 1 month (December 2019) clustering of patients with fever only or fever and other symptoms [6].
(21) Was there one or more explosive outbreaks from the Huanan Seafood Wholesale Market in Wuhan with indicators of a point source origin?
(22) Was there a high concentration of the COVID-19 virus in the environment (incriminated Huanan Seafood Wholesale Market)?
(23) Were there the existence of biological risk and a biological threat in the Hubei province for the COVID-19 outbreak?

## 2 Methods

A literature review was performed in the database MEDLINE for the period from January 1, 2000 to October 31, 2020, to obtain the necessary data for a retrospective analysis of the COVID-19 outbreak. The period from January 1, 2000 to October 31, 2020 was reviewed because of the possibility to find the appropriate scientific method for investigating the pandemic origin and very similar epidemiological event. The guiding question of the literature review was the origin of the COVID-19 pandemic.

The types of reviewed literature were original research articles and review articles published in the English language in peer-reviewed journals.

Criteria for inclusion in the review were the following keywords: outbreak, pandemic, COVID-19, outbreak origin, outbreak differentiating, case fatality rate, mortality, contact tracing, SARS-CoV-2, and China.

The time period in which the research was carried out was from July 1 to December 31, 2020.

Two search strategies were used for the article selection and accordingly a number of articles were studied. The first search strategy for the article selection was focused on the type and the origin of the COVID-19 outbreak. Query box “All fields” was used, because of its comprehensiveness. Other query boxes gave an unsatisfied number of references.

Step one: COVID-19 AND outbreak origin = 1101 references; terms to the query box: All fields.

Step two: COVID-19 AND outbreak origin AND outbreak differentiating = 38 references; terms to the query box: All fields.

Step three: COVID-19 AND outbreak origin AND outbreak differentiating AND China = 14 references; terms to the query box: All fields.

The second search strategy for the article selection was focused on the key outbreak parameters and the original name of the virus from the COVID-19 outbreak. Query box “TEXT WORD” was used, because it gave a satisfied number of references.

Step one: COVID-19 AND case fatality rate AND mortality = 291 references; terms to the query box: TEXT WORD.

Step two: COVID-19 AND contact tracing = 1300 references; terms to the query box: TEXT WORD.

Step three: SARS-CoV-2 AND outbreak origin = 0 references; terms to the query box: TEXT WORD.

First, large groups of the articles were rejected for further consideration after reading the articles’ titles. After reading the abstracts of the articles, the second most numerous group of articles were rejected for further consideration. The third group of articles was rejected after reading the articles’ methods and results. The fourth group of articles was rejected after reading the entire article. Finally, the fifth group of articles was rejected after reading and mutually comparing with other articles (criteria were article scientific informativeness and scientific reliability).

There are three methods for outbreak differentiating. The Radosavljevic–Belojevic method has two parts. The first part for early orientation and differentiation of unusual epidemiological events (UEEs) was applied and is shown in Table 1. Characteristics of this part of the method are described in refs [7,8]. After this first orientation about the type of COVID-19 outbreak, the second part of the method is subtle and detailed differentiation of the four possible UEE scenarios: natural outbreak of a known disease, natural outbreak of a new or re-emerging disease, outbreak by an accidental release of a pathogen, and outbreak by a deliberate delivery of a biological agent (described in refs [1,9]) is shown in Table 2. In both the parts of the method, indicators were scored with 1 if they were present in the outbreak, and scored with 0 if indicators were not present in the outbreak. Also, the Radosavljevic–Belojevic method was the most suitable to answer questions from Section 1.

**Table 1:** Scoring of the COVID-19 outbreak according to the model of Radosavljevic and Belojevic [7,9] for differentiation between natural, accidental, and deliberate outbreak caused by new or re-emerging pathogen

No.	Epidemiological/infectiological indicators	Score
1	Unusual/atypical disease/manifestation (symptoms/signs) or unexpected fulminant course of disease in humans and/or animals	1
2	Failure of patient to respond to usual therapy or illness in a population (human and animal) despite immunizations	1
3	Several unusual/unexplained syndromes coexisting in the same case without any other explanation	1
4	Sudden unexplainable increase in the number of cases or deaths in human and/or animal population	1
5	Morbidity and/or mortality higher than expected	1
6	Clustering of patients with fever and/or fever and respiratory symptoms and/or lymphadenopathy	1
7	Disease identified in the region for the first time ever or again after a long period of time	1
8	Disease with an unusual/atypical seasonal distribution	1
9	Simultaneous occurrence of epidemics and/or epizootics	1
10	Explosive epidemics and/or epizootics with indicators on a point-source origin	1
11	Disease with an unusual geographic distribution	1
12	Occurrence of a non-endemic (imported) or previously eradicated disease	0
13	Epidemiological data suggesting a common exposure	1
14	Simultaneous epidemics and/or epizootics occur at different locations	1
15	Total score	13

1 = high probability of a deliberate or accidental outbreak, 0 = low probability of a deliberate or accidental outbreak, assessment of scores: 1–4 probably natural outbreak, 5–9 possibly deliberate or accidental outbreak, 10–14 probably deliberate or accidental outbreak.

**Table 2:** Assessment of the COVID-19 pandemic origin in 2019–2020 by differentiation scoring for a natural outbreak of a disease (NE), a natural outbreak of a new or re-emerging disease (NR), an outbreak by an accidental release of a pathogen (AR), and a deliberate outbreak (DO)

Parameter	NE	NR	AR	DO
**Perpetrator/source of infection/reservoir of pathogen**				
Sophistication		N/A	1	
Motivation		N/A	0	
Intention		N/A	0	
Intelligence		N/A	0	
Secrecy		N/A	0	
Number of perpetrators		N/A	0	
Number of sources of infection/reservoirs		1	1	
Accessibility to sources of agent/pathogen		1	1	
Accessibility to targets/population at risk		1	1	
**Biological agent/pathogen**				
A category*		0	0	
B category*		0	0	
C category*		0	0	
Emerging pathogen		1	1	
Amount of the available agent/pathogen		1	1	
**Means/media of delivery/factors of transmission**				
Air		1	1	
Food		1	1	
Water		0	0	
Fomites		1	1	
Vectors		0	0	
Biological ammunition		0	0	
Delivery systems		0	0	
Dispersion systems/mechanism of release		0	0	
**Target/susceptible population at risk**				
Intelligence		0	1	
Secrecy		1	1	
Personal control		1	1	
Control of means/media of delivery/factors of transmission		1	1	
Physical protection		1	1	
Protection by chemoprophylaxis		0	0	
Protection by immunoprophylaxis		0	0	
Importance of target/population at risk		0	0	
Location of target/population at risk		0	1	
Number of people in a target/population at risk		0	1	
Distribution of people in a target/population at risk		0	1	
Total	—	12	17	—

*CDC classification, 0 = low probability, 1 = high probability, N/A = not applicable/no data, – = eliminated from further consideration, total scores: 0–8 = lowly probable type of outbreak (TO), 9–16 = possible TO, 17–24 = highly probable TO, 25–33 = certain TO.

Two other methods for outbreak differentiating are not appropriate for the COVID-19 outbreak, because they are focused on differentiating between natural and deliberate outbreaks. They do not have scenarios for accidental outbreaks and outbreaks caused by a new or re-emerging pathogen and that was the reason for not including them in this analysis [10,11].

## 3 Results

In Table 1, the total score indicates that the COVID-19 outbreak was UEE with the features of probably accidental or deliberate epidemics.

Indicator 1. The clinical picture of the COVID-19 deviated from the expected one in pneumonia [12,13,14,15]. In a significant number of convalescents, senses of taste and smell were lost (scored with 1).

Indicator 2. Patients with the COVID-19 disease did not respond to the usual antibiotic and/or antiviral therapy (scored with 1).

Indicator 3. The outbreak was characterized by bilateral pneumonia, frequent requirement of artificial ventilation, and heart damage [13]. Significant number of patients lost senses of taste and smell and had extreme fatigue. The unexplainable rapid increase in the number of such patients was alarming for the wider scientific medical community and for the China government (scored with 1).

Indicator 4. Mortality and case fatality rate of pneumonia were higher than expected [16,17]. There are no data about animals sick or dead due to the COVID-19 virus (scored with 1).

Indicator 5. See indicator 4. There were no data about China, and because of the very high percentage of “undocumented” pneumonia patients it was not possible to calculate morbidity rate even approximately, but surely it was higher than expected (scored with 1) [18].

Indicator 6. See indicator 4. There were especially family clustering cases of pneumonia (scored with 1) [19].

Indicator 7. The outbreak was caused by a new pathotype of the coronaviruses, the COVID-19 virus. Chinese authorities informed WHO about new pathogen and new disease on December 31, 2019, and sent a worldwide sequence of the viral genome on January 12, 2020 (scored with 1) of pneumonia cases [4].

Indicator 8. After 1 year of the outbreak start and spread worldwide, obviously there was no seasonal distribution of the COVID-19 (scored with 1).

Indicator 9. There were no data about epizootics. Outbreak expanded to pandemic, so there were a lot of simultaneous epidemics worldwide (scored with 1).

Indicator 10. The epidemic was explosive and with indicators of a point-source origin because in a few weeks from the local outbreak it became pandemic comprising all continents and nearly all states and territories (scored with 1).

Indicator 11. Each pandemic (except flu) has an unusual geographical distribution.

Indicator 12. COVID-19 was a new disease, not non-endemic (imported) or previously eradicated disease, so this indicator was scored with 0.

Indicator 13. Common exposure to potential sources of pathogens was strictly forbidden, logically it was common exposure at the start of the outbreak and numerous times after that (scored with 1).

Indicator 14. See indicator 9.

The total score was 13 out of 14 points, which meant it was probably the artificial (deliberate or accidental) epidemic. Because of that, a method for subtle and detailed differentiation of the four possible UEE scenarios, namely natural outbreak of a known disease, natural outbreak of a new or re-emerging disease, outbreak by an accidental release of a pathogen, and outbreak by a deliberate delivery of a biological agent, was used.

### 3.1 Deliberate outbreak scenario

COVID-19 outbreak may not be a biological attack (BA) because of several issues:

(1) There is no BA which became pandemic in a short time;
(2) SARS-CoV-2 is not a bioweapon because of low lethality;
(3) If any BSL-4 laboratory tries to produce bioweapon, it must have already or simultaneously produce “antidote” (vaccine, serum, or drugs);
(4) There is no information about any country with BSL-4 laboratory, which has experimented with coronaviruses as a bioweapon.

### 3.2 Natural outbreak scenario

COVID-19 outbreak may not be a naturally originated outbreak because of several issues:

(1) There is no scientific evidence that the COVID-19 virus exists in any animal;
(2) There is no scientific evidence that the COVID-19 virus transfers from any animal to a human;
(3) In natural epidemics, sources/reservoirs of infection may be discovered by usual epidemiological and microbiological routine investigations, and there are no tendencies to keep themselves unknown. For over 1 year from the start of the COVID-19 outbreak, nobody reported scientifically detailed initial phases (source; reservoir of the outbreak; way of transmission of the first case; who, when, and how exactly the people were infected; etc.) of the course of the COVID-19 outbreak, and why?
(4) In natural outbreaks, the number and distribution of sources of infection are related to the incubation period and period of disease communicability of pneumonia [5]. There is no detailed and logical order of such data from the beginning of the COVID-19 outbreak.

Consequently, two types of outbreaks, BA and naturally originated outbreak, are eliminated from further consideration.

### 3.3 Natural outbreak of a new or re-emerging disease

#### 3.3.1 Sources/reservoirs of the infection

Animals as a source/reservoir of the outbreak of pneumonia have no next indicators: sophistication, motivation, intention, intelligence, secrecy, and number of perpetrators (Table 2) [1,9]. But parameters such as number of sources/reservoirs of virus, accessibility to sources of the virus, and accessibility to targets surely exist in the case of animals as a source/reservoir of the outbreak and scored 1 for each parameter.

#### 3.3.2 Biological agent vs pathogen

COVID-19 virus is an emerging agent like SARS virus, Ebola virus, MERS virus, Swine flu virus, and Avian flu virus. The epidemic was explosive (many people were infected in a brief period), which meant that the pathogens were released in large amounts during a short time. It was not possible that a couple of bats or even a whole flock of them heat-treated might infect so many people in urban Wuhan population with pneumonia [20,21,22]. It was much more possible that bat hunters and traders became first diseased and clearly traced the source of the outbreak. Because of the abovementioned reasons these two parameters scored 1 each.

#### 3.3.3 Means/media of delivery vs factors of transmission

Air, food, and fomites are proved ways of transmission of pneumonia and each of them scored 1 [23,24,25].

### 3.4 Target vs susceptible population at risk

#### 3.4.1 Intelligence

The first cases and even more clusters were not recognized and the infection spread throughout China and worldwide (scored with 0).

#### 3.4.2 Secrecy

Chinese authorities did not provide relevant information about this UEE and reported pneumonia to WHO on December 31, 2019 [26]. There was no international scientific cooperation about the origin of the outbreak (source/reservoir of the infection, trace of possible infected human contacts, or infected animals) [27]. During the SARS pandemic, Chinese authorities hid information about epidemics for 6 weeks and during that time epidemic “advanced” to pandemic [1]. So, 1 point for secrecy.

#### 3.4.3 Personal control

Personal control was very rigorous (wearing face masks, physical distancing among people, isolation of areas even with several million inhabitants, advice for hand hygiene, intensified other hygienic measures and their control, and intensified recognition of potential infected or diseased people (measuring temperature). This parameter scored 1.

#### 3.4.4 Control of means/media of delivery/factors of transmission

According to the available data, the control of means/media of delivery/factors of transmission was carried out rigorously and was very successful (isolation – “quarantine,” and disinfection of commonly used and frequently used surfaces). Therefore, this parameter is scored with 1.

#### 3.4.5 Physical protection measures

Physical protection measures for the population at risk were not in place during the initial phase of the outbreak. Later, such measures were strictly applied (see Sections 3.4.3 and 3.4.4) [27]. Consequently, this parameter was scored with 1.

#### 3.4.6 Protection by chemoprophylaxis and/or by immunoprophylaxis

There was protection neither by chemoprophylaxis nor by immunoprophylaxis, so both parameters were scored with 0.

#### 3.4.7 Importance of target/population at risk

There was no evidence that the so-called “hard targets” or “soft targets” were aimed, and this parameter is scored with 0.

#### 3.4.8 Number of people in target/at risk

Densely populated metropolitan areas like Wuhan were in favor of a deliberate outbreak, but not of a natural outbreak of a new or re-emerging disease or an accidental outbreak. So, this parameter is scored with 0.

#### 3.4.9 Location and distribution of people in target/at risk

No special targets (e.g., military, political, economic, or cultural) could be identified. Rural areas (caves) as natural habitats for bats, or jungles for pangolins, but not metropolitan areas like Wuhan with 11 million densely populated inhabitants, are preferred locations for the outbreak focus in the case of a natural outbreak or a new or re-emerging disease. So, these two parameters are scored each with 0.

### 3.5 Accidental release outbreak scenario

#### 3.5.1 Sources/reservoirs of the infection

In the case of the accidental outbreak scenario parameter, sophistication must be scored with 1. Parameters such as motivation, intention, intelligence, secrecy, and number of perpetrators are scored each with 0. But parameters such as number of sources/reservoirs of the virus, accessibility to sources of virus, and accessibility to targets surely existed in the accidental outbreak scenario and scored each with 1.

#### 3.5.2 Biological agent vs pathogen

COVID-19 virus is an emerging agent (see Section 3.3). In the case of the accidental release outbreak scenario, the staffs were not probably conscious of the repeated release of the COVID-19 virus from the laboratories. How long it was unknown, but surely the virus was released in a large amount during a short time. Because of the abovementioned reason, this parameter is scored with 1.

#### 3.5.3 Means/media of delivery vs factors of transmission

Air, food, and fomites are proved ways of transmission and each of them scored with 1 [23,24,25].

### 3.6 Target vs susceptible population at risk

#### 3.6.1 Intelligence

Some Chinese doctors were “pressed” to withdraw their reports about new and unusual pneumonia. Even more, in the case of accidental pathogen release, authorities were very quickly informed about the accident and this parameter is scored with 1.

#### 3.6.2 Secrecy

See Section 3.4.2.

#### 3.6.3 Personal control

See Section 3.4.3.

#### 3.6.4 Control of means/media of delivery/factors of transmission

See Section 3.4.4.

#### 3.6.5 Physical protection measures

See Section 3.4.5 [28].

#### 3.6.6 Protection by chemoprophylaxis and/or by immunoprophylaxis

See Section 3.4.6.

#### 3.6.7 Importance of target/population at risk

See Section 3.4.7.

#### 3.6.8 Number of people in target/at risk and location and distribution of people in target/of people at risk

Laboratories with BSL-4 are usually located near the big cities and university centers because of the highly qualified manpower who lived there, such as a densely populated metropolitan area like Wuhan that was in favor of an accidental outbreak.

Also, such laboratories are usually located in large scientific and/or medical centers with many people employed, frequenting hundreds in the same building.

So, these three parameters were scored each with 1.

#### 3.6.9 Score interpretation

Final scores of the differentiation method suggest that the high probable cause of COVID-19 outbreak was by an accidental release of a new COVID-19 virus from the Wuhan Institute of Virology. This was probably attributable to a technical accident and/or negligent violation of hygienic norms in the laboratory facility.

## 4 Discussion

From the very start of the COVID-19 outbreak, there was a lack of scientific data about the pathogen source and the mode of its transmission [29]. Bats, probably heat-treated, as the source of the outbreak were questionable.

Analysis of the unusual COVID-19 epidemic in 2019/2020 by the two-part method (Radosavljevic–Belojevic method) for differentiation between natural outbreak, accidental outbreak, deliberate outbreak, and outbreak caused by a new or re-emerging pathogen showed that this epidemic was an accidental outbreak caused by the new pathogen, probably attributable to a technical accident and/or negligent violation of hygienic norms in the laboratory facility.

The method used is strictly focused on specific outbreak characteristics important for outbreaks differentiating and in total has 47 indicators – outbreak features. There is a general agreement between the results of the two parts of the method. The first part almost completely (with 13 out of 14 indicators) indicates a deliberate or accidental outbreak. Indicators 1, 2, 3, 7, 8, and 11 are of essential importance for determining an artificial (accidental or deliberate) outbreak nature. Their scoring with each one as contributing key clues presents additional evidence for the artificial nature of the outbreak. So, the question arises concerning the COVID-19 epidemic: “What was it, actually?”

The second part for subtle and detailed outbreak differentiation, clearly at the start declines natural outbreak scenario and deliberate outbreak scenario. The final results show 12 points for the outbreak scenario caused by the new or re-emerging pathogen and 17 points for the accidental outbreak scenario.

This method clarified the German *Escherichia coli* outbreak in 2011, indicating an accidental outbreak scenario. Also, the method is retrospectively used and published in reputable publications, in other “famous” epidemics (Ameritrax in 2001, Sverdlovsk outbreak in 1979, Kosovo tularemia outbreak in 1999/2000, and Swine flu in 2009/2010). Additionally, the method was first tested on several dozen outbreaks before publishing (data not shown).

The new hybrid and chimeric COVID-19 virus combines features of known viruses and differs in its genetic and pathogenic features from known coronaviruses. There are over 1400 species of bats, at least 3200 distinct coronaviruses that infect bats, and numerous articles about bats as possible/probable culprit sources of the COVID-19 virus [2]. But, there is no scientific proof of bats infected with the COVID-19 virus. Even more, incriminated kinds of bats live in other continents and there are no data about the COVID-19 virus in those bats [2,15]. Actually, except being suspicious, we do not know the source/reservoir of the COVID-19 epidemic.

A long-lasting exposure to the pathogen for several weeks, until authorities’ interventions became effective, indicating that an accidental release was very possible in the COVID-19 epidemic [15]. With very obscure data about outbreak origin (source/reservoir of the outbreak, course of the outbreak in the first week(s)), suspicion about intentional hiding of data increases.

If the bats from the fish market were reservoirs for the COVID-19 virus (fish market was closed on January 1, 2020), authorities should know who brought and sold them on the market [29,30]. Authorities could find natural habitat and reservoirs for the COVID-19 disease tracing those people [29,30]. Furthermore, without solving the source/reservoir of the COVID-19 outbreak, it is not possible to prevent similar outbreak again worldwide.

In conclusion, this study suggests that the COVID-19 outbreak was a consequence of an accidental release of a new COVID-19 virus, probably during the technical accident and/or negligent violation of hygienic norms in the laboratory facility. Accordingly, permanent laboratory personnel education, maximal hygienic norms in the laboratory facilities, and urgent international scientific cooperation are necessary to effectively prevent and resolve similar possible outbreaks. Further epidemiological, microbiological, and forensic analyses are needed to clarify the COVID-19 outbreak origin. Detection of people with SARS-CoV-2 antibodies in Northern Italy probably would show that because of very intensive business relationships (production of modern clothes, shoes, and handbags) between Wuhan and Milano, the pandemic began much earlier and had a very clandestine start [31].

## References

[1] Radosavljevic V. A new method of differentiation between a biological attack and other epidemics. In: Hunger I, Radosavljevic V, Belojevic G, Rotz LD, editors. Biopreparedness and public health. Heidelberg: Springer; 2013. p. 17–32.

[2] Sun K, Viboud C. Impact of contact tracing on SARS-CoV-2 transmission. Lancet Infect Dis. 2020;20(8):876–7. 10.1016/S1473-3099(20)30357-1. PMC7185949 32353350

[3] Worldmeter. Available from: https://www.worldmeters.info/coronavirus/countries/; 2000 (Accessed 29 December 2020).

[4] Burki T. Outbreak of coronavirus disease 2019. Lancet Infect Dis. 2020;20(3):1018–9. 10.1016/S1473-3099(20)30076-1. PMC7128260 32078809

[5] Radosavljevic V, Banjari I, Belojevic G, editors. Defence against bioterrorism: methods for prevention and control. 1st ed. Heidelberg: Springer; 2018.

[6] World Health Organization. Origin of SARS-CoV-2; 2020 March 26. Available from: https://apps.who.int/iris/handle/10665/332197

[7] Radosavljevic V, Belojevic G. Unusual epidemilogical event – new model for early orientation and differentation between natural and deliberate outbreak. Public Health. 2012;126(1):77–81. 10.1016/j.puhe.2011.11.006. 22136700

[8] Radosavljevic V, Finke E-J, Belojevic G. Escherichia coli O104:H4 outbreak in Germany – clarification of the origin of the epidemic. Eur J Public Health. 2015;25(1):125–9. 10.1093/eurpub/cku048. 24736168

[9] Radosavljevic V, Finke E-J, Belojevic G. Analysis of the Escherichia coli O104:H4 outbreak in Germany in 2011 by a differentiation method for unusual epidemiological events. Cent Eur J Public Health. 2016;24(1):9–15. 10.21101/cejph.a4255. 27070964

[10] Grunow R, Finke E-J. A procedure for differentiating between the intentional release of biological warfare agents and natural outbreaks of disease: its use in analyzing the tularemia outbreak in Kosovo in 1999 and 2000. Clin Microbiol Infect. 2002;8(8):510–21. 10.1046/j.1469-0691.2002.00524.x. 12197873

[11] Dembek ZF, Kortepeter MG, Pavlin JA. Discernment between deliberate and natural infectious disease outbreaks. Epidemiol Infect. 2007;135(3):353–71. 10.1017/S0950268806007011. PMC2870591 16893485

[12] Bagcchi S. Mysterious pneumonia in China. Lancet Infect Dis. 2020;20(2):173. 10.1016/S1473-3099(20)30011-6. PMC7128697 32006509

[13] Topol EJ. COVID-19 can affect the heart. Science. 2020;370(6515):408–9. 10.1126/science.abe2813. 32967937

[14] Wu JT, Leung K, Bushman M, Kishore N, Niehus R, de Salazar PM, et al. Estimating clinical severity of COVID-19 from the transmission dynamics in Wuhan, China. Nat Med. 2020;26(4):506–10. 10.1038/s41591-020-0822-7. PMC7094929 32284616

[15] Wu Y-C, Chen C-S, Chan Y-J. The outbreak of COVID-19: An overview. J Chin Med Assoc. 2020;83(3):217–20. 10.1097/JCMA.0000000000000270. PMC7153464 32134861

[16] Joy M, Hobbs FDR, McGagh D, Akinyemi O, de Lusignan S. Excess mortality from COVID-19 in an English sentinel network population. Lancet Infect Dis. 2020;21(4):e74. 10.1016/S1473-3099(20)30632-0. PMC7402647 32763192

[17] Rajgor DD, Lee MH, Archuleta S, Bagdasarian N, Chye Quek S. The many estimates of the COVID-19 case fatality rate. Lancet Infect Dis. 2020;20(7):776–7. 10.1016/S1473-3099(20)30244-9. PMC7270047 32224313

[18] Li R, Pei S, Chen B, Song Y, Zhang T, Yang W, et al. Substantial undocumented infection facilitates the rapid dissemination of novel coronavirus (SARS-CoV-2). Science. 2020;368(6490):489–93. 10.1126/science.abb3221. PMC7164387 32179701

[19] Jing Q-L, Liu M-J, Zhang Z-B, Fang L-Q, Yuan J, Zhang A-R, et al. Household secondary attack rate of COVID-19 and associated determinants in Guangzhou, China: a retrospective cohort study. Lancet Infect Dis. 2020;20(10):1141–50. 10.1016/S1473-3099(20)30471-0. PMC7529929 32562601

[20] Malaiyan J, Arumugam S, Mohan K, Radhakrishnan GG. An update on the origin of SARS‐CoV‐2: Despite closest identity, bat (RaTG13) and pangolin derived coronaviruses varied in the critical binding site and O‐linked glycan residues. J Med Virol. 2021;93(1):499–505. 10.1002/jmv.26261. PMC7361880 32633815

[21] Morens DM, Breman JG, Calisher CH, Doherty PC, Hahn BH, Keusch GT, et al. The origin of COVID-19 and why it matters. Am J Trop Med Hyg. 2020;103(3):955–9. 10.4269/ajtmh.20-0849. PMC7470595 32700664

[22] Latinne A, Hu B, Olival KJ, Zhu G, Zhang L, Li H, et al. Origin and cross-species transmission of bat coronaviruses in China. Nat Commun. 2020;11(1):4235. 10.1038/s41467-020-17687-3. PMC7447761 32843626

[23] Karia R, Gupta I, Khandait H, Yadav A, Yadav A. COVID-19 and its modes of transmission. SN Compr Clin Med. 2020;2:1798–801, Epub 2020 Sep 1. 10.1007/s42399-020-00498-4 PMC7461745 32904860

[24] van Doremalen N, Bushmaker T, Morris DH, Holbrook MG, Gamble A, Williamson BN, et al. Aerosol and Surface Stability of SARS-CoV-2 as Compared with SARS-CoV-1. N Engl J Med. 2020;382(16):1564–7. 10.1056/NEJMc2004973. PMC7121658 32182409

[25] Zhang Y, Chen C, Zhu S, Shu C, Wang D, Song J, et al. Isolation of 2019-nCoV from a stool specimen of a laboratory-confirmed case of the coronavirus disease 2019 (COVID-19). China CDC Weekly. 2020;2(8):123–4. 10.46234/ccdcw2020.033. PMC8392928 34594837

[26] Thorp HH. The costs of secrecy. Science. 2020;367(6481):959. 10.1126/science.abb4420. 32108091

[27] Thanh HN, Van TN, Thu HNT, Van BN, Thanh BD, Thu HPT, et al. Outbreak investigation for COVID-19 in northern Vietnam. Lancet Infect Dis. 2020;20(5):535–6. 10.1016/S1473-3099(20)30159-6. PMC7158986 32145188

[28] Lau H, Khosrawipour V, Kocbach P, Mikolajczyk A, Schubert J, Bania J, et al. The positive impact of lockdown in Wuhan on containing the COVID-19 outbreak in China. J Travel Med. 2020;27(3):taaa037. 10.1093/jtm/taaa037. PMC7184469 32181488

[29] Zhang X, Chen X, Zhang Z, Roy A, Shen Y. Strategies to trace back the origin of COVID-19. J Infect. 2020;80(6):e39–40. 10.1016/j.jinf.2020.03.032. PMC7141644 32277970

[30] Yu WB, Tang G-D, Zhang L, Corlett RT. Decoding evolution and transmissions of novel pneumonia coronavirus (SARS-CoV-2) using the whole genomic data. Zool Res. 2020;41(3):247–57. 10.24272/j.issn.2095-8137.2020.022. PMC7231477 32351056

[31] Apolone G, Montomoli E, Manenti A, Boeri M, Sabia F, Hyseni I, et al. Unexpected detection of SARS-CoV-2 antibodies in the prepandemic period in Italy. Tumori J. 2020. First published online: November 11. 10.1177/0300891620974755. PMC8529295 33176598

